# Drop-out rate among patients treated with omalizumab for severe asthma: Literature review and real-life experience

**DOI:** 10.1186/s12890-016-0290-5

**Published:** 2016-08-25

**Authors:** M. Caminati, G. Senna, G. Stefanizzi, R. Bellamoli, S. Longhi, F. Chieco-Bianchi, G. Guarnieri, S. Tognella, M. Olivieri, C. Micheletto, G. Festi, E. Bertocco, M. Mazza, A. Rossi, A. Vianello, on behalf of North East Omalizumab Network study group, C. Barp, L. Bonazza, M. A. Crivellaro, A. Dama, G. Donazzan, G. Idotta, C. Lombardi, M. Nalin, C. Pomari, M. Schiappoli

**Affiliations:** 1 Asthma Center and Allergy Unit, Verona General and University Hospital, Verona, Italy; 2 Respiratory Pathophysiology Division, University-City Hospital of Padua, Padua, Italy; 3 Department of Cardiologic, Thoracic and Vascular Sciences, University of Padua, Padua, Italy; 4 Respiratory Unit, Orlandi General Hospital, Bussolengo, Verona, Italy; 5 Unit of Occupational Medicine, Verona General and University Hospital, Verona, Italy; 6 Respiratory Unit, Mater Salutis Hospital, Legnago, Verona, Italy; 7 Pulmonary Unit, Verona University and General Hospital, Verona, Italy; 8 Respiratory pathology Unit, Arzignano General Hospital, Vicenza, Italy; 9 Pulmonary Unit, Pordenone General Hospital, Pordenone, Italy

**Keywords:** Drop-out, Adherence, Severe asthma, Omalizumab

## Abstract

**Background:**

In patients with asthma, particularly severe asthma, poor adherence to inhaled drugs negatively affects the achievement of disease control. A better adherence rate is expected in the case of injected drugs, such as omalizumab, as they are administered only in a hospital setting. However, adherence to omalizumab has never been systematically investigated. The aim of this study was to review the omalizumab drop-out rate in randomized controlled trials (RCTs) and real-life studies. A comparative analysis was performed between published data and the Italian North East Omalizumab Network (NEONet) database.

**Results:**

In RCTs the drop-out rate ranged from 7.1 to 19.4 %. Although the reasons for withdrawal were only occasionally reported, patient decision and adverse events were the most frequently reported causes. In real-life studies the drop-out rate ranged from 0 to 45.5 %. In most cases lack of efficacy was responsible for treatment discontinuation. According to NEONet data, 32 % of treated patients dropped out, with an increasing number of drop outs observed over time. Patient decision and lack of efficacy accounted for most treatment withdrawals.

**Conclusions:**

Treatment adherence is particularly crucial in patients with severe asthma considering the clinical impact of the disease and the cost of non-adherence. The risk of treatment discontinuation has to be carefully considered both in the experimental and real-life settings. Increased knowledge regarding the main reasons for patient withdrawal is important to improve adherence in clinical practice.

## Background

Adherence is usually defined as the extent to which the patient’s use of medication matches the prescribed regimen. Poor adherence has a critical relevance in the management of various chronic diseases as it may negatively affect treatment outcomes and result in increased hospitalizations, morbidity, and mortality [1]. In bronchial asthma the achievement of disease control is closely related to adherence. It has been extensively demonstrated that an irregular drug intake markedly affects patient’s quality of life, as it is responsible for an increased risk of nocturnal awakenings and impairment in routine daily activities, such as exercise and sports [2].

Lack of adherence is very common, particularly in chronic conditions [3–7]. In fact, the treatment discontinuation rate ranges from 20 to 40 % for acute illnesses and from 30 to 60 % for chronic diseases. Preventive treatments are associated with a non-adherence rate of up to 80 % [8]. As far as asthma is concerned, it is well known that about 50 % of patients are non-adherent. The issue becomes even more relevant in specific age groups such as children, adolescents and elderly [9].

A number of factors, including fear of treatment-related side effects, poor perception of symptoms, belief in alternative/complementary medicine but also complex treatment regimens, illness-related factors, inconvenience, and social background, may account for poor adherence, which has a very high social cost [1]. In the United States, irregular drug intake among patients with hypertension is responsible for 89 premature deaths every year [10]. It is estimated that annually $US100 billion which is spent on unnecessary or preventable hospitalizations related to poor adherence could be saved [11].

Many of the above mentioned variables have been described as determinants of poor adherence to asthma treatments. Also, poor awareness of the need for treatment even in the absence of symptoms and steroids fear are two main reasons for treatment withdrawal in asthmatic patients [12]. On the other side, caregivers themselves have to face some limitations such as difficulties in patients follow-up scheduling and time constraints, which may hamper patient’s adherence support [9].

A univocal and standardized tool for evaluation of adherence is lacking. Although many methods are currently available none of them can be considered as the gold standard [1]. Another controversial aspect concerns the definition of “acceptable adherence”. In some large studies, an adherence rate greater than 80 % has been considered satisfactory [1] but a general consensus about this issue has not been reached.

In bronchial asthma evaluation of adherence is even more difficult as treatment is mainly based on inhaled drugs. In this case, an objective adherence evaluation may rely on different tools but due to many limitations with these tools their use in routine clinical practice is not suitable. Electronic devices are accurate but are also quite expensive [13]; therefore recording of pharmacy refills can be considered as a more affordable option but they are less accurate. Patients usually deny any lack of adherence, even in severe asthma [14].

Treatment discontinuation is one of the most relevant aspects of adherence, as it leads to major consequences. From this perspective, drop-out rate can be considered to be a surrogate marker of adherence. In randomized controlled trials (RCTs) a high drop-out rate can weaken the final results [15], and in the real-life setting it is related to preventable patient impairments and unnecessary costs. Furthermore, the detection and quantification of drop-out rate in a daily clinical setting is a complex issue, as regular follow-up for all patients is not always possible. Patients requiring treatment with injected drugs are more easily monitored, as treatment administration requires medical supervision. This is the case with omalizumab, an anti-immunoglobulin E (IgE) monoclonal antibody for the treatment of severe asthma. It must be administered in a hospital setting once or twice a month, according to the patient’s total IgE level [16]. Thus, treatment discontinuation can be easily detected and considered as a consistent marker of adherence.

To our knowledge, the treatment discontinuation rate among patients undergoing omalizumab treatment has never been systematically investigated as a primary outcome. The present study aimed to review the drop-out rate and describe the most common reasons for patient withdrawal in RCTs and real-life studies published up to December 2014. A comparative analysis between published data and an Italian database, the North East Omalizumab Network (NEONet), was also conducted.

## Methods

### Search strategy

A complete search of the Cochrane Central Register of Controlled Trials (CENTRAL) in the Cochrane Library, MEDLINE and PubMed up to December 2014 was carried out. The search strategy retrieved citations containing the subject heading “omalizumab” and was restricted to randomized, double-blind, placebo-controlled trials and “real life studies” for severe allergic asthma in patients aged ≥18 years old. The keywords used were: omalizumab, asthma, controlled studies, randomized trial, real life studies, pragmatic studies. All retrieved studies were restricted to the English language.

### Italian North East Omalizumab Network (NEONet) database analysis

A retrospective analysis of the NEONet database was carried out. Details about the Network and the data collecting methods are provided elsewhere [17]. In brief, NEONet is a non-profit project approved by the local ethics committee and involves 19 Allergy and Respiratory Referral Centres for Severe Asthma located in the North-East region of Italy. It aims to collect an extensive amount of clinical data on patients undergoing omalizumab treatment in a real-life setting and provide some new insights concerning current unmet needs (e.g. impact of omalizumab treatment on lung function and on asthma comorbidities, long-term follow-up of treated patients, adherence, non-responders profile, optimal treatment duration). The participating clinicians enter anonymous coded data into a shared limited-access web platform.

### Drop-out evaluation

The drop-out rate and the most common reasons for treatment discontinuation were evaluated, if reported, in RCT, in “real life studies” and in the NEONet database. Reasons for withdrawal were categorized as follows: patient’s decision, lack of efficacy, adverse event, clinical efficacy, and other causes. With regard to the NEONet database, “lack of efficacy” and “clinical efficacy” were defined according to the GETE (global evaluation of treatment effectiveness) Questionnaire [18], which was completed by physicians for every patient. GETE is a five-point scale: 1 is excellent (complete control of asthma), 2 is good (marked improvement), 3 is moderate (discernible, but limited improvement), 4 is poor (no appreciable change), and 5 is worsening. Number 4 and 5 corresponded to “lack of efficacy” and number 1 and 2 corresponded to “clinical efficacy”.

### Statistical analysis

Two-sample t-test was used to compare the variables in Table 4. Two-proportion z-test was used to analyze the differences between mean drop-out rates and reasons. All tests have been performed with a significance level of 5 %. A logistic regression analysis was provided in order to verify the association between drop-out rate and treatment duration. R software has been used.

## Results

### Randomized controlled studies (RCTs)

As of December 2014, seven RCTs have been published (Table 1) [16, 19–24]. Overall, 1719 patients were included, with a slightly higher prevalence of females. Across the studies, the mean age ranged from 37.5 to 43.7 years. The drop-out rate, which was reported in all the analyzed studies, ranged from 7.1 to 19.4 %. However, reasons for withdrawal were not always reported. Patient’s decision accounted for the majority of drop outs in four of the selected studies [16, 20, 21, 24]; whereas in two studies adverse events were the main cause of treatment discontinuation [19, 22]. No data were available with regard to the timing of drop-outs after the initiation of treatment.

**Table 1:** Overall dropout rate and main reasons for treatment discontinuation in RCT

Author [ref]	N	duration (months)	Mean ± SD age, years	M/F Ratio	Drop-out rate (%)	Patient decision (%)	Lack of efficacy (%)	Adverse events (%)	Other Causes (%)
Busse et al. 2001 [16]	268	7	39.3	0.63	7.1	4.1	0.37	0.74	1.49
Soler et al. 2011 [23]	274	7	40.0	1.06	6.9	1.09	1.09	NR	NR
Holgate et al. 2004 [21]	126	8	41.1	0.55	8.7	8.7	NR	NR	NR
Ayres et al. 2004 [19]	206	12	37.5	0.39	7.3	NR	NR	7.3	NR
Vignola et al. 2004 [24]	209	7	38.3 ± 14.7	0.92	8.1	8.1	NR	NR	NR
Humbert et al 2005 [22]	209	7	43.4 ± 13.3	0.48	12.2	NR	NR	4.5	NR
Hanania et al. 2011 [20]	427	12	43.7 ± 14.3	0.63	19.4	11.00	NR	3.74	4.68

*F* female, *M* male, *NR* not reported, *RCT* randomized controlled trials, *SD* standard deviation

### Real-life studies

As of December 2014, 19 real-life studies have been published on the use of omalizumab in severe asthma (Table 2) [25–42]. A total of 13,466 patients were included: compared with RCTs the age range was broader in real-life studies (29.5 to 49.9 years) with the inclusion of patients aged almost 10 years younger and 6 years older than RCT patients. The mean study duration was longer in real-life studies.

**Table 2:** Overall dropout rate and main reasons for treatment discontinuation in real-life studies

Author [ref]	N	duration (months)	Mean ± SD age, years	M/F Ratio	Drop-out rate (%)	Patient decision (%)	Lack of efficacy (%)	Adverse events (%)	Other Causes (%)
Molimard et al. 2008 [37]	146	12	46.5 ± 13.5	0.57	30.6	1.4	19	5.4	4.8
Brusselle et al. 2009 [29]	158	12	48.1 ± 17.1	0.85	45.5	10.1	13.3	12	10.1
Korn et al. 2009 [34]	280	5	43.9 ± 16.3	1.45	32.5	NR	14.28	NR	NR
Bavbek et al. 2010 [27]	18	6	41.8 ± 11.2	0.63	0	NR	NR	NR	NR
Cazzola et al. 2010 [30]	142	24	49.6 ± 4.1	1	8.5	2.11	1.4	1.4	3.5
Tzortzaki et al. 2012 [39]	60	26	54.0 ± 14.0	0.66	0	NR	NR	NR	NR
Wittchen et al. 2012 [42]	53	24	48.3 ± 13.7	1	NR	NR	NR	NR	NR
Vennera M et al. 2012 [40]	266	15	51.0 ± 13.7	0.45	18.7	5.6	10.5	2.6	NR
Lafeuille et al. 2012 [35]	644	24	49.9 ± 14.2	0.69	NR	NR	NR	NR	NR
Eisner et al. 2012 [32]	4969	12	44.5 ± 16.6	0.56	NR	NR	NR	NR	NR
Chen et al. 2013 [31]	4970	48	44.5 ± 16.6	0.56	NR	NR	NR	NR	NR
Grimaldi-Bensouda et al. 2013 [33]	374	36	49.7 ± 14.6	0.58	NR	NR	NR	NR	NR
Barnes et al. 2013 [26]	136	36	41.3 ± 14.5	0.46	NR	NR	NR	NR	NR
Maselli et al. 2013 [36]	26	6	29.6 ± 18.7	1.6	0	NR	NR	NR	NR
Group 1^a^									
Maselli et al. 2013 [36]	26	24	34.0 ± 17.6	NR	0	NR	NR	NR	NR
Group 2^b^									
Braunstahl et al. 2013 [28]	943	12	45.0 ± 15.5	NR	16.6	8.4	NR	NR	8.2
Özgür et al. 2013 [38]	26	6	47.6 ± 13.9	0.23	0	NR	NR	NR	NR
Vieira et al. 2014 [41]	15	6	45.6 ± 10.8	0.15	26.66	6.66	NR	20.00	NR
Ancochea et al. 2014 [25]	214	12	48.2 ± 17.7	0.43	7.9	4.2	2.3	1.9	NR

*F* female, *M* male, *NR* not reported, *RCT* randomized controlled trials, *SD* standard deviation

^a^Patients with IgE levels above 700 IU/mL

^b^Patients with IgE levels less or equal to 700 IU/mL

In the 13 studies that reported the drop-out rate, it ranged from 0 to 45.5 %. No patients discontinued treatment in four of these studies [27, 36, 38, 39]. There was notable variability in the reasons given for discontinuing treatment across the different studies. Lack of efficacy was the most common reason for treatment discontinuation in most studies [29, 34, 37, 40]. Patient decision to discontinue treatment was the main reason for drop outs in two studies [25, 28] and adverse events were the most frequent reason for withdrawal in one study [37].

### Comparison between RCTs and Real-life studies

Table 3 provides a direct comparison of drop-out rates and reasons between RCTs and Real-life studies. Overall, drop-out rate in Real-life studies is significantly higher. The proportion of patients who discontinued omalizumab due to a lack of efficacy was significantly bigger in real-life studies than in RCTs. On the opposite patient’s decision and adverse events have more relevance in RCTs in comparison with Real-life studies.

**Table 3:** Comparison of drop-out rate mean values and reasons between RCTs and Real-life studies

	RCT	Real-life	*p*-value
Drop-out	11.65 %	17.50 %	0.0000
Patient decision	59.72 %	35.01 %	0.0000
Lack of efficacy	0.69 %	23.53 %	0.0000
Adverse events	22.92 %	12.04 %	0.0011
Other causes	116.67 %	29.41 %	0.0016

### NEONet database

The NEONet database included 221 patients. As shown in Table 4, among them 70 (32 %) dropped out; under treatment population and drop-outs did not significantly differ in terms of age, gender and mean treatment duration. Treatment discontinuation was more common amongst females (64 %). Patient decision accounted for most of the withdrawals (49 %), followed by a lack of efficacy (26 %). Within the group of patients dropping-out for “onset of contraindications”, pregnancy was the reason in all the cases. As far as adverse events is concerned, 3 cases of generalized urticarial have been described; arthralgia and myocarditis have been recorded in two other cases.

**Table 4:** Overall drop-out rate and main reasons for treatment discontinuation in the NEONet database

Patient population (*n* = 221)	Drop-out patients (70, 32 %)	Patients under treatment (151, 38 %)	*p-value*
Males, n (%)	25 (35.71)	68 (45.03)	0.0959
Females, n (%)	45 (64.29)	83 (54.97)	
Age-years, mean (SD)	46.79 (14.82)	47.44 (13.11)	0.4904
Treatment duration-months, mean (SD)	27.69 (20.94)	27.54 (22.96)	0.4992
*Reason for drop-out, n (%)*			
Lack of efficacy	18 (26)		
Patient’s decision discontinuation	34 (49)		
Efficacy	4 (6)		
Adverse events (local or systemic reactions)	5 (7)		
Onset of contraindications	6 (8)		
Patient moved to another referral center	3 (4)		

*NEONet* North East Omalizumab Network, *SD* standard deviation

Table 5 provides a comparative overview of NEONet database and published Real-life studies in terms of drop-out rates and reasons. The overall drop-out rate was significantly higher in NEONet database. Patient’s decision as a cause of dropping out showed the same trend. On the opposite drop-out rates due to lack of efficacy and adverse events do not significantly differ between NEONet and published Real-life studies.

**Table 5:** Comparison of drop-out rate mean values and reasons between NEONet database and published Real-life studies

	NEONet	Real-life	*p*-value
Drop-out	31.67 %	17.50 %	0.0000
Patient decision	48.57 %	35.01 %	0.0160
Lack of efficacy	25.71 %	23.53 %	0.3475
Adverse events	7.14 %	12.04 %	0.1176
Other causes	18.57 %	29.41 %	0.0318

**Fig. 1:**
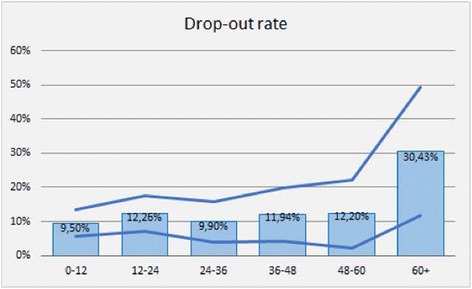
Drop-out rates in different treatment duration time intervals (NEONet database; *n* = 221). The blue lines indicate the 95 % Confidence Interval (CI)

In our study population the proportion of drop-outs does not significantly change in different treatment duration time intervals, as described in Fig. 1, which also shows the 95 % Confidence Interval (CI) (blue lines). Furthermore, the logistic regression analysis confirms the lack of association between time and drop-out rate (*p* = 0.96).

## Discussion

This review of publications on omalizumab in severe asthma demonstrated that there is a wide variability in both the drop-out rate and the reasons for discontinuing treatment. Drop-out rates appeared to be the lowest in RCTs—possibly because these studies are conducted under rigorously controlled conditions. In contrast, real-life studies, which are more closely aligned with routine clinical practice, cited markedly higher drop-out rates of up to 46 %. The NEONet database was more in line with the data from real-life studies with reported dropout rates of 32 %. Lack of efficacy was cited as one of the most common reasons for treatment discontinuation in both real-life studies and the NEONet database, while patient decision and adverse events primarily contributed to the drop-out rates observed in RCTs.

Poor treatment adherence is a well-known unmet need in patients with asthma. This is particularly the case with inhaled drugs [2, 13, 43]. Data regarding adherence patterns in patients treated with omalizumab are limited and the evidence is weakened by methodological differences across the studies [44]. However, despite the reported drop-out rates, adherence to omalizumab appears to be slightly higher than that observed with other anti-asthmatic drugs [45, 46]. Therefore, omalizumab therapy has been proposed as an alternative for patients with poorly controlled asthma for whom adherence does not improve with conventional interventions [46]. One possible explanation is that compared with oral or inhaled treatments, omalizumab is regularly administered in a hospital setting under direct medical supervision thereby improving treatment adherence. Conversely, subcutaneous allergen immunotherapy, which is also regularly administered in a hospital setting to patients with respiratory allergy [47], is characterized by a lower adherence rate in comparison to omalizumab [6, 48]. Of note, this immunotherapy is indicated in mild to moderate asthmatics with less severe symptoms [47]. On this basis, it could be argued that disease severity can positively affect adherence to treatment. A non-adherence rate of 44 % in asthmatics with steroid-dependent asthma has been reported [14]. Furthermore, a recent observational study on omalizumab adherence identified a lower pre-bronchodilator percentage of predicted forced expiratory volume in 1 s (FEV_1_) as an independent predictor of good adherence [44].

Treatment discontinuation unrelated to medical reasons represents the major drawback of non-adherence. It implies there are preventable direct and indirect costs affecting both patient quality of life and health systems resources [49]. To the best of the authors’ knowledge the drop-out rate among patients undergoing omalizumab treatment has never been systematically investigated as a primary outcome.

Overall, a lower rate of treatment discontinuation with a narrow range was observed in RCTs. This finding is to be expected if the setting of experimental studies is taken into account. In fact, the RCT protocol typically mandates regular patient assessment and a strict follow-up schedule, often with a shorter duration of follow-up in comparison with real-life studies. All of these factors may account for a lower withdrawal rate, which is also a methodological requirement in order to strengthen the final results [15, 50]. Surprisingly, the reasons for patient drop outs were not reported in some RCTs [21, 24]. However, adverse effects and patient decision were responsible for most drop outs across the reviewed studies [25, 28, 29, 34, 37, 40]. RCT protocols are usually demanding for patients and withdrawal due to inconvenience is not unexpected [48]. As far as adverse effects are concerned, RCT protocols include strict and careful monitoring of potential treatment-related adverse events that more frequently results in patient exclusion from the study than in the real-life setting [50, 51].

In the real-life studies, drop-out prevalence was characterized by a marked variability, ranging from 0 % in four studies up to 45 % in the remaining studies. Although the reasons for drop-outs were sporadically reported, in most cases lack of efficacy was responsible for treatment discontinuation. The proportion of patients who discontinued omalizumab due to a lack of efficacy was significantly higher in real-life studies than in RCTs. The different patient selection process may provide a possible explanation. In fact, patients’ enrolment in RCTs relies on strict inclusion and exclusion criteria, which differs from the real-life setting.

A recent review from our group [51] has highlighted that sensitization to a perennial allergen is missing in more than 20 % of patients undergoing omalizumab treatment, despite being included among the prescription criteria established by the European Medicines Agency (EMA) [52]. However, non-responders have also been described among patients matching all the EMA prescription criteria [17, 28], and the efficacy of omalizumab in non-atopic asthma is also supported by the literature [53]. Patient selection, particularly in the field of biological drugs for asthma, still represents a challenge [54, 55]. The relevance of a number of biomarkers has been recently investigated and still fosters current research. The poor specificity of many molecules and the complex relationship between symptoms, exacerbations, response to drugs and underlying inflammation hampers the identification of univocal and standardized biomarkers predictive of clinical response [56, 57]. Such biomarkers are still lacking for omalizumab and for current and upcoming biological treatments for severe asthma [55, 58].

Nevertheless, patient selection is one of the most important aspects in managing biological drugs as they target a very specific mechanism in the pathophysiologic picture of the disease [58, 59].

Omalizumab has a good safety profile, both in the experimental and real-life setting. Only three studies, two RCTs [19, 22] and one real-life study [37], reported adverse events as the main cause of treatment discontinuation, without any significant differences in terms of drop-out rate. Of note, a local reaction at the injection site was the commonest adverse event. This finding suggests that tolerability is an important issue and consequently it has to be carefully considered; as evidenced with other treatments, it can significantly affect adherence [60]. Therefore, clinicians should discuss tolerability issues with their patients as part of a strategy aimed at improving adherence.

The results from the NEONet database were similar to those reported in published studies, although the overall drop-out rate seems to be higher in our study population. For project’s policy, Referral Centers included in the NEONet collaboration are requested to strictly and regularly follow-up the patients, thus under this perspective our clinical practice is more similar to a RCT setting than to a pure real-life one. It may provide an explanation for our finding. However drop-out rates due to lack of efficacy and adverse events do not significantly differ between NEONet and published Real-life studies. The population sample was smaller in comparison with other real-life studies, however it is quite homogeneous, as the patients live in the same geographical area, and the centers share the same diagnostic work-up and patient selection criteria [17]. In the NEONet population, patient decision was the most common reason for dropping out. Although several reasons can influence patient choice, inconvenience may play the most relevant role [6, 14]. In fact, the need for regular administration of omalizumab in hospital once or twice per month can strongly affect treatment adherence, as it has many implications such as work-absenteeism and economic burden. Under this perspective, patient’s perception of clinical efficacy as well as lack of efficacy, has a crucial relevance as it may weaken the motivation of the patient for continuing treatment.

Interestingly, in our study population treatment length does not seem to affect drop-out rate. In fact, the proportion of drop-outs is similar in all the treatment duration time intervals (Fig. 1). Apparently the drop-out rate in the last interval is higher, but the small sample size in that range may account for this effect, as shown in the graph by the CI bars. Furthermore, the logistic regression analysis confirms the lack of association between time and drop-out rate (*p* = 0.96). Whether the length of treatment impacts on the adherence rate is not easy to evaluate in the published studies, due to the great variability in terms of study duration [25–42]. However the ideal treatment duration, as well as the identification of biomarkers that are accurate in predicting the clinical response are still lacking [58, 59]. In fact, lack of efficacy, similarly to the published Real life studies [25–42], accounted for 26 % of drop-outs among NEONet patients, despite all patients being fully matched with the current prescription criteria. In the real-life setting, many patient-related variables, such as smoking habits, comorbidities, and multi-drug treatments, may affect treatment efficacy and effectiveness [51, 61], even though prescription criteria are verified. In this scenario patient’s education, in terms of awareness of the treatment and its implications, has an even more relevant role in preventing drop-outs and generally supporting adherence [62, 63].

Some limitations of our work deserve to be highlighted. Two variables potentially affecting the drop-out rate, lung function at baseline and prescribed medications other than Omalizumab, have not been extensively analyzed. In the case of the first determinant, few data are available in literature, however a lower forced expiratory volume in 1 s (FEV_1_) has been described as an independent predictor of good adherence [44]. Concerning the published studies included in the present review [16, 19–42], a systematic analysis of the lung function and its impact on drop-out rate is not easy relying on the available information, affected by great variability, or not mentioned at all. In our dataset analysis, GETE questionnaire for the evaluation of clinical efficacy indirectly includes the impact of treatment on lung function. As lack of efficacy is one of the main drop-out reasons, it could be hypothesized that a poor lung function at baseline, maintained during the treatment, may act as a determinant of poor adherence.

A great variability, or the lack of detailed information, also regards the prescribed medications other than Omalizumab [16, 19–42] and hampers an extensive analysis of this further drop-out determinant. The scenario is even more complex if we consider the amount of drugs prescribed for comorbidities. Such analysis is out of the aim of our paper and requires an adequately sized population sample. However, according to the literature adherence to omalizumab appears to be slightly higher than that observed with other anti-asthmatic drugs, independently of other medication prescribed at the same time [45, 46].

A second limitation of our work relates to the study design itself; in real-life observational studies it is difficult to avoid or properly assess bias, and conclusions are not easily applicable across a generalized population. Furthermore, often only a descriptive analysis has been provided. Nevertheless, to our knowledge treatment discontinuation rate has never been systematically investigated as a primary outcome in a real-life setting and awareness of the most common reasons for patient withdrawal may help in finalizing some practical suggestions to improve adherence in routine clinical practice.

## Conclusion

In conclusion, the risk of treatment discontinuation is a significant drawback for omalizumab therapy and this warrants consideration when prescribing. The reasons for dropping out have to be carefully taken into consideration when planning specific long-term strategies in order to prevent treatment withdrawal.

## Abbreviations

EMA: European Medicines Agency
FEV_1_: Forced expiratory volume in 1 s
GETE: Global evaluation of treatment effectiveness
IgE: Immunoglobulin E
NEONet: North East Omalizumab Network
RCT: Randomized clinical trial
